# Construction and validation of a deterioration model for elderly COVID-19 Sub-variant BA.2 patients

**DOI:** 10.3389/fmed.2023.1137136

**Published:** 2023-04-13

**Authors:** Yinyan Wu, Benjie Xiao, Jingjing Xiao, Yudi Han, Huazheng Liang, Zhangwei Yang, Yong Bi

**Affiliations:** ^1^Department of Neurology, Translational Research Institute of Brain and Brain-Like Intelligence, Shanghai Fourth People’s Hospital Affiliated to Tongji University School of Medicine, Shanghai, China; ^2^Shanghai Key Laboratory of Anesthesiology and Brain Functional Modulation, Shanghai, China; ^3^Monash Suzhou Research Institute, Suzhou Industrial Park, Suzhou Jiangsu, China; ^4^Medical Department, Shanghai Fourth People’s Hospital, School of Medicine, Tongji University, Shanghai, China; ^5^Affiliated Zhoupu Hospital, Shanghai University of Medicine and Health Siences, Shanghai, China; ^6^Translational Research Institute of Brain and Brain-Like Intelligence, Shanghai Fourth People’s Hospital Affiliated to Tongji University School of Medicine, Shanghai, China

**Keywords:** coronavirus, COVID-19, deterioration model, prognosis, prediction

## Abstract

**Rationale:**

COVID-19 pandemic has imposed tremendous stress and burden on the economy and society worldwide. There is an urgent demand to find a new model to estimate the deterioration of patients inflicted by Omicron variants.

**Objective:**

This study aims to develop a model to predict the deterioration of elderly patients inflicted by Omicron Sub-variant BA.2.

**Methods:**

COVID-19 patients were randomly divided into the training and the validation cohorts. Both Lasso and Logistic regression analyses were performed to identify prediction factors, which were then selected to build a deterioration model in the training cohort. This model was validated in the validation cohort.

**Measurements and main results:**

The deterioration model of COVID-19 was constructed with five indices, including C-reactive protein, neutrophil count/lymphocyte count (NLR), albumin/globulin ratio (A/G), international normalized ratio (INR), and blood urea nitrogen (BUN). The area under the ROC curve (AUC) showed that this model displayed a high accuracy in predicting deterioration, which was 0.85 in the training cohort and 0.85 in the validation cohort. The nomogram provided an easy way to calculate the possibility of deterioration, and the decision curve analysis (DCA) and clinical impact curve analysis (CICA)showed good clinical net profit using this model.

**Conclusion:**

The model we constructed can identify and predict the risk of deterioration (requirement for ventilatory support or death) in elderly patients and it is clinically practical, which will facilitate medical decision making and allocating medical resources to those with critical conditions.

## Highlights

- Emerging SARS-CoV-2 BA.2.2 threatens patients’ lives all over the world.
- A new model to estimate the risk of deterioration for patients inflicted by Omicron variants was built using a combination of blood biomarkers.
- The model we constructed can identify and predict the risk of deteriorationin of elderly patients and it is clinically practical.

## Introduction

The COVID-19 pandemic has continued to overwhelm healthcare systems worldwide. The outbreak of Omicron sub-variant BA.2 in Shanghai in March 2022 has resulted in heavy medical burden to the healthcare system and economy recession (1). Due to the effective vaccination and variation of the virus, clinical characteristics of Omicron inflicted patients are totally different from those who were infected by COVID 19 when it broke out in Wuhan in 2020 (2). Effective triage of patients presenting to the hospital for risk of progressive deterioration is crucial to inform clinical decision making and to facilitate effective resource allocation, including hospital beds, critical care resources, and targeted drug therapies (3). Since the majority of Omicron sub-variant BA.2 patients show mild symptoms and a low mortality rate, early identification of subgroups of patients at a high risk of death or deterioration and requiring ventilation enables precise delivery of treatments.

Many multivariable clinical prognostic models for patients with COVID-19 have been developed to predict adverse outcomes, such as mortality or clinical deterioration (4–7). The deterioration model developed by the International Severe Acute Respiratory and emerging Infections Consortium Coronavirus Clinical Characterization Consortium (ISARIC4C) study (4) has combined 11 predictors to predict clinical deterioration and achieved a good clinical utility (c-statistic = 0.77), but the population included both confirmed and suspected COVID-19 patients and the missing data in the study led to the difficulty in integrating important variables into the model, such as D-dimer, which may cause the negligence of the clinical characteristics. Fang et al. (5) established a prognostic model based on 10 variables, which achieved a good prognostic value (AUC = 0.89), but it did not include patients aged ≥89, and it had a decent percentage of missing data. COVID-GRAM (6) established a deterioration model which included 1,590 Chinese patients and combined 10 indicators to build an online free risk calculator, which had a good prognostic value (AUC = 0.88), but the mean age of the included population was 48.9 years, and it was based on the COVID-19 outbreak in 2020 in Wuhan, China. The end-stage liver disease (MELD)(7) score model included 4,213 COVID-19 confirmed patients, but the model was also based on data collected during the outbreak of COVID-19 in 2020. Due to the difference in multiple respects between the two outbreaks (8), there is a surging demand to build a new model to estimate the risk of deterioration for patients inflicted by Omicron variants.

In the present study, a large cohort of COVID-19 patients with confirmed Omicron variant BA2.2 was utilized to develop a prognostic model for in-hospital clinical deterioration (requirement for ventilatory support or death) and its efficacy was validated in a separate cohort.

## Methods

### Study population

This research represented a single-center, retrospective study on COVID-19 patients admitted to Shanghai Fourth People’s Hospital affiliated to Tongji University between 22nd March and 17th June, 2022. During this time, COVID-19 patients were transferred to our hospital which was utilized exclusively to admit patients who had a positive SARS-CoV-2 reverse transcriptase polymerase chain reaction (RT-PCR) of Omicron sub-variant BA.2. Patients of different severity were all admitted for treatment in order to reduce negative conversion time of the SARS-CoV-2 RNA. Diagnosis of SARS-CoV-2 infection was based on the Guidelines for Diagnosis and Treatment of Novel Coronavirus Pneumonia (9th version) (9). The inclusion criteria included: patients were over 65 years old, and were admitted to the hospital for positive COVID-19 for the first time, and all the regarding data were obtained within the first 24 h in the hospital, and they reached the final endpoints during the hospitalization (death, requirement of ventilatory support, or discharged).

### Outcome

A composite primary outcome of in-hospital clinical deterioration, comprising initiation of ventilatory support (non-invasive ventilation, invasive mechanical ventilation) and death.

### Predictors

Electronic medical records (EMRs) were used to collect patients’ characteristics, including clinical features, symptoms, comorbidities, laboratory findings, treatments (including antiviral or anti-inflammatory drugs), and outcomes (discharged or death or requirement of ventilatory support), by following the standardized approach to each variable definition (10). All information was documented on a standardized record form.

Those variables were divided into routinely available and laboratory ones. Routinely available predictors included age, gender, comorbidities which were defined on the basis of the modified Charlson comorbidity index (11). Laboratory measurements included C-reactive protein (CRP), interleukin-6 (IL-6), procalcitonin (PCT), white blood cell count (WBC), neutrophil count, lymphocyte count, neutrophil/lymphocyte ratio (NLR), monocyte count, platelets (PLT), hemoglobin (HB), RDW-CV (red blood cell distribution width-coefficient variation), Red blood cell count (RBC), Albumin (ALB), Albumin/globulin (A/G), creatinine (Crea), blood urea nitrogen (BUN), aspartate aminotransferase (AST), alanine aminotransferase (ALT), total bilirubin (TBIL), γ-glutamyltransferase (GGT), glomerular filtration rate (GFR), prothrombin time (PT), thrombin time (TT), activated partial thromboplastin time (APTT), Fibrinogen (FIB), international normalized ratio (INR). Only the first results of measured predictors within 24 h of admission were included. These predictors were selected on the basis of literature describing their close association with COVID-19 prognosis (12). Predictors were excluded if they had incomplete information in order to minimize the deviation of the regression coefficient.

This study was conducted by complying with the principles of the Declaration of Helsinki. The associated Ethics Committee of Shanghai Fourth People’s Hospital approved the study and waived the need for informed consent. The methodology of the study followed the guidelines for transparent reporting of a multivariable prediction model for individual prediction or diagnosis (TRIPOD) (13).

### Statistical analysis

#### Prediction model development

All patients were randomly assigned to a training cohort and a validation cohort (7:3). The predictive model and nomogram were constructed in the training cohort based on baseline characteristics as well as results of the first laboratory tests after admission, and they were then validated in the validation cohort.

Feature selection was performed using the least absolute shrinkage and selection operator (LASSO) regression method with the R package “glmnet” to identify the relative importance of each feature. Those important predictors were then entered into the logistic stepwise regression analysis to minimize the variable range. Finally, the coefficients of the logistic regression model were used to construct the prediction model using R “glm” function. After that, the R “rms” package was used to build the Nomograms (14).

A common problem should be taken into consideration when comparing laboratory data between institutions is that laboratory values are highly dependent on the methodologies used, and data normalization is needed. In general, results of laboratory tests have normal ranges that enclose 95% of values in a healthy population. When the laboratory values were beyond the testing ability of the lower or upper values of the normal range, we recommended to use the laboratory’s upper or lower limits as the value input.

#### Model performance assessment

Performance of nomograms was evaluated using discrimination [the area under the receiver operating characteristic curve (AUROCC)], calibration (calibration plots), and clinical applicability [decision curve analysis (DCA) and clinical impact curve analysis (CICA)] in R. During the internal validation of the nomogram, the total score of each patient in the testing cohort and their corresponding progression probability were calculated according to the established nomogram. Clinical utility was analyzed using the decision curve analysis and clinical impact curve analysis in the R “rmda” package (15). It was used to measure the net benefit using the prediction model in clinical practice which were compared between the treat-all and the treat-none modes. The concept of net benefit can be hard to understand, it could be interpreted as a hypothetical scenario where the prognostic index was used to decide whether a patient needed to be treated. All analyses were done in R (version 4.1.2).

## Results

**Figure 1:**
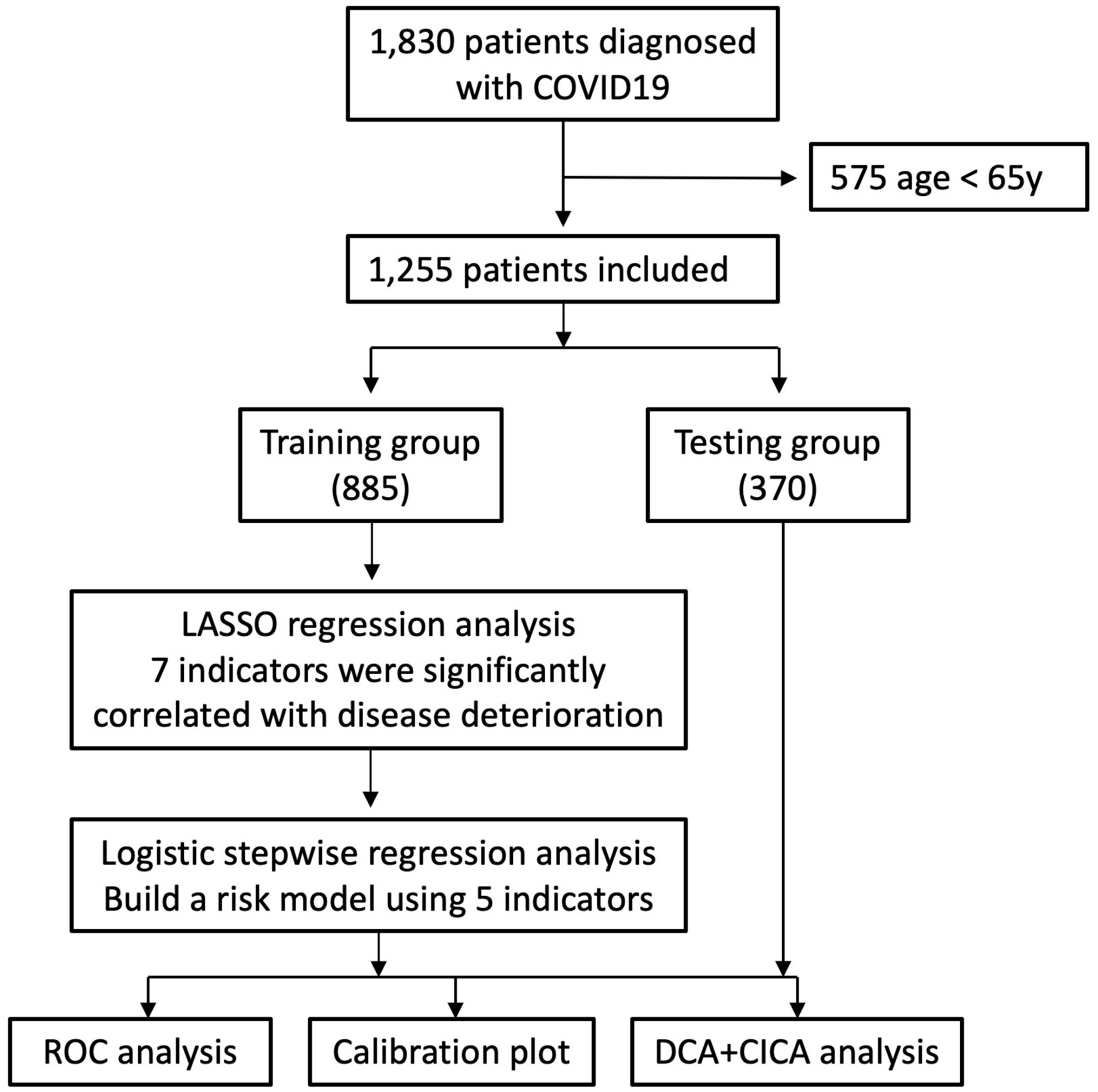
Flowchart of the study showing the recruitment process and data analysis as well as modeling.

The selection process of the study population was illustrated in Figure 1. A total of 1,830 patients with COVID-19 were enrolled and 575 excluded due to their age younger than 65. Baseline characteristics between the training cohort and the validation cohort were shown in Tables 1, 2. The average time from PCR confirmation to admission was 2 days. Nearly half of them had hypertension (49.5%). The majority of these elderly patients showed mild to moderate symptoms, with a small proportion of them having severe and critical symptoms. Most patients received the Paxlovid (84.5%) and anticoagulation (71.0%) treatments. The proportion of deterioration rate was 11.6%. No difference was observed in above variables between the training cohort and the validation cohort.

**Table 1:** Clinical characteristics of the training and the testing cohorts.

	Total (*N* = 1,255)	Training cohort (*N* = 885)	Testing cohort (*N* = 370)	*p* value
Age,median(IQR),yr	82(73,89)	82(73,88)	82(73,89)	0.37
Sex, *M*, *n*(%)	514(40.9%)	365(41.2%)	149(40.3)	0.80
PCR confirmation to hospital admission median (IQR),d	2(1,5)	2(1,5)	3(1,5)	0.34
Comorbidity, *n*(%)				
Hypertension	622(49.5%)	421(47.6%)	201(54.3%)	0.034
Diabetes mellitus	276(22.0%)	181(20.5%)	95(25.7%)	0.05
Atrial fibrillation	68(0.1%)	47(5.3%)	21(5.7%)	0.90
Myocardia infarcion	18(1.4%)	14(1.6%)	4(1.1%)	0.67
Parkinson disease	29(2.3%)	20(2.3%)	9(2.4%)	0.10
dementia	78(6.2%)	53(6.0%)	25(6.8%)	0.70
Ischemic stroke/TIA	260(20.7%)	173(19.5%)	87(23.5%)	0.13
Peripheral atrial disease	10(0.8%)	7(8.0%)	3(8.0%)	0.10
Severity-A, *n*(%)				
No symptom	49(3.9%)	35(4.0%)	14(3.8%)	0.10
Mild	561(44.7%)	399(45.1%)	162(43.8%)	0.72
Median	511(40.7%)	353(39.9%)	158(42.7%)	0.39
Severe	102(8.13%)	75(8.5%)	27(7.3%)	0.56
Critical	28(2.23%)	19(2.1%)	9(2.4%)	0.92
Treatment, *n*(%)				
Paxlovid	1,021(84.5%)	717(84.1%)	304(85.6%)	0.55
Anticoagulation	863(71.0%)	616(72.2%)	247(69.6%)	0.39
Antibacterial drugs	359(29.7%)	250(29.3%)	109(30.7%)	0.68
Traditional Chinese medicines	717(59.4%)	50(59.0%)	214(60.3%)	0.72
Steroid	147(12.2%)	100(11.7%)	47(13.2%)	0.52
Outcome, *n* (%)				
Ventilation support /death	146(11.6%)	101(11.4%)	45(12.2%)	0.78

Continuous variables of skewed distribution were showed as median [interquartile range, IQR] and compared with Mann–Whitney test, categorical variables were presented as numbers (percentage) and compared by the chi-square test. Definition of abbreviations: IQR = interquartile range; Severity-A = severity on admission. TIA, transit ischemic stroke.

**Table 2:** Laboratory findings at study entry.

	Total (*N* = 1,255)	Training cohort (*N* = 855)	Testing cohort (*N* = 370)	*p* value
Routin blood tests				
Whiet cell count (3.5–9.5)	5.13(3.97,6.78)	5.15(4.01,6.75)	5.04(3.86,6.89)	0.57
Neutrophils (1.8–6.3)	3.20(3.97,6.78)	3.18(2.25,4.59)	3.35(2.22,4.64)	0.55
Lymphocytes (1.1–3.2)	1.19(0.85,1.61)	1.21(0.87,1.66)	1.14(0.80,1.53)	0.01
NLR	2.59(1.65,4.60)	2.52(1.61,4.47)	2.85(1.82,4.94)	0.03
Monocytes (0.1–0.6)	0.43(0.32,0.57)	0.43(0.32,0.56)	0.43(0.32,0.57)	0.71
Platelets (125–350)	167.0 (131.0,214.0)	169.0(133.0,215.0)	161.0(125.0,202.8)	0.04
hemoglobin (115–150)	124 (110,134)	125(110,134)	123(112,135)	0.89
RDW-CV (11-16)	13.40(13.90,14.20)	13.40(12.90,14.20)	13.50(12.90,14.20)	0.93
RBC (3.8–5.1)	4.13(3.69,4.51)	4.12(3.68,4.51)	4.15(3.71,4.51)	0.68
Inflammatory marker				
CRP (0-6 mg/L)	10.49(3.66,31.87)	10.16(3.38,31.68)	11.04(4.50,32.00)	0.28
IL-6(<6.6)	34.66(17.21,132.30)	36.47(17.47，132.3)	33.03(16.38，127.83)	0.52
procaicltonin (<0.5)	0.02(0.02,0.08)	0.023(0.02,0.078)	0.022(0.02,0.087)	0.98
Coagulation test				
D-dimmer (<0.5)	0.70(0.40,1.40)	0.70(0.40,1.43)	0.69(0.41,1.2)	0.97
PT (9.4–12.5)	11.30(10.70,12.10)	11.30(10.60,12.10)	11.20(10.70,12.00)	0.90
TT (13–21)	14.30(13.50,15.20)	14.3(13.55,15.25)	14.3(13.50,15.10)	0.30
APTT (23.5–40.7)	29.60(27.70,31.80)	29.60(27.70,31.70)	29.65(27.70,32.00)	0.45
FIB (2–5)	4.12(3.70,4.57)	4.13(3.69,4.59)	4.11(3.75,4.55)	0.77
INR	1.03(0.97,1.10)	1.03(0.96,1.10)	1.02(0.97,1.09)	0.94
Renal and liver function				
Albumin (40–55)	38.47(35.52,41.15)	38.45(35.46,41.12)	38.58(35.84,41.27)	0.61
A/G (1.2–2.4)	1.70(1.45,1.95)	1.71(1.45,1.97)	1.69(1.45,1.93)	0.71
TBIL (<23)	11.07(8.22,15.52)	11.13(8.19,15.47)	10.93(8.28,15.56)	0.77
GGT (10–60)	20.96(15.34,31.08)	20.81(15.28,32.03)	21.40(15.42,29.59)	0.80
ALT (9–50)	15.77(11.33,23.97)	15.83(11.33,24.21)	15.67(11.46,23.47)	0.65
AST (15–40)	24.43(19.35,32.57)	24.52(19.48,32.57)	24.32(19.06,32.53)	0.47
BUN (3.6–9.5)	6.02(4.69,8.07)	5.96(4.68,8.10)	6.165(4.75,8.045)	0.54
Cr (57–111)	62.50(50.90,80.30)	62.40(50.10,80.40)	62.55(52.70,80.10)	0.46
GFR (90–120)	96.00(74.00,120.00)	97.0(74.0,121.0)	93.50(73.0,118.75)	0.34

Continuous variables of skewed distribution were showed as median [interquartile range, IQR] and compared with Mann–Whitney test. CRP,C-reactive protein; IL-6,Interleukin-6; WBC,white blood cell count; NLR, neutrophil/lymphocyte ratio; RDW-CV, red blood cell distribution width-coefficient variation; RBC, Red blood cell count; A/G, Albumin/globulin; Crea, creatinine; BUN, blood urea nitrogen; AST, aspartate aminotransferase; ALT, alanine aminotransferase; TBIL,total bilirubin; GGT, γ-glutamyltransferase; GFR, golomeruar filtration rate; PT, prothrombin time; TT, thrombin time; APTT, activated partial thromboplastin time; FIB, Fibrinogen; INR,international normalized ratio Reference ranges for each blood measurement are included in parentheses. All cell counts are ×10^9^/L.

### Model development and internal validation

**Figure 2:**
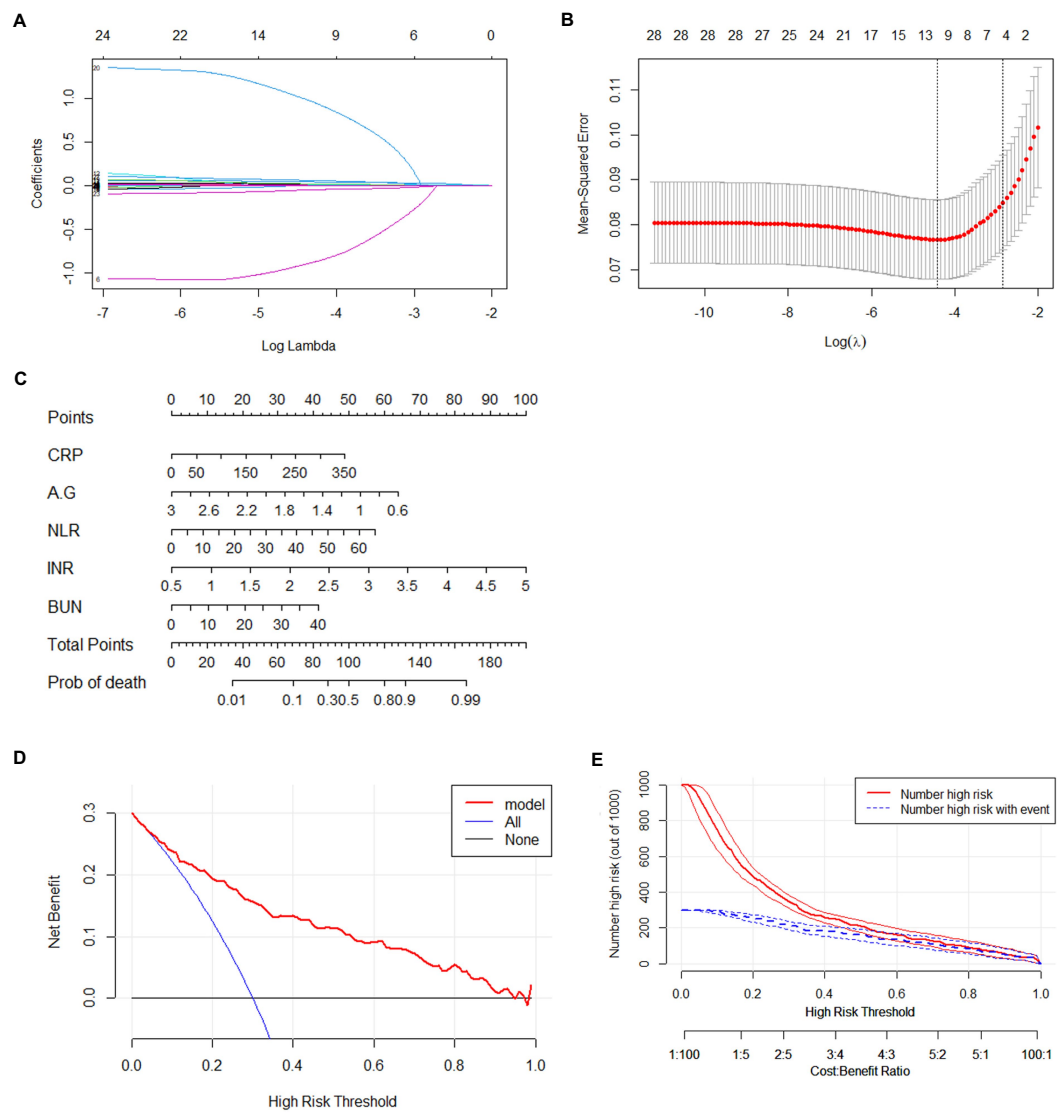
Construction of prediction nomogram in patients with COVID-19. **(A)** LASSO coefficient profiles (y-axis) of the 45 features. The lower x-axis indicated the log lamda, the top x-axis has the average numbers of predictors. **(B)** Identification of the optimal penalization coefficient (λ) in the LASSO model was performed *via* 3-fold cross validation based on minimum criteria. **(C)** Nomogram predicting the deteriorated COVID-19 probability in patients with COVID-19 infection was plotted. **(D)** Decision curve compares the net clinical benefits of three scenarios in predicting the deteriorated COVID-19 probability: treat- all (red line), treat- none (horizontal solid black line), and screen based on the nomogram (red line). **(E)** Clinical impact curve of the nomogram plot the number of COVID-19 patients classified as high risk (red line), and the number of cases classified high risk with deterioration at each high risk threshold (blue line). CRP, C-reactive protein; NLR, neutrophil/lymphocyte ratio; A/G: Albumin/globulin; BUN: blood urea nitrogen; INR: international normalized ratio; CI, confidence interval.

A total of 44 features were collected from each patient in the training cohort which consisted of 855 patients and 29 continuous variables entered for LASSO regression analysis (Figures 2A,B). The remaining 8 important variables were then registered with the Logistic regression for deeper selection. Results of 855 patients in the training cohort showed that CRP, A/G, NLR, INR, BUN were predictive factors for clinical deterioration of COVID-19 (Table 3). Prediction models were then built using the coefficients form the above results.

**Table 3:** Final multivariable model in training dataset.

Variable	β coefficients (95%CI)	Odds ratio (95%CI)	*p* value
CRP	0.0097(0.0053,0.01)	1.0098(1.0054,1.01)	<0.001***
A/G	−1.85(−2.62,1.08)	0.158(0.073,0.34)	<0.001***
NLR	0.061(0.015, 0.11)	1.06(1.02, 1.11)	0.0102*
INR	1.54(0.58,2.51)	4.69(1.79,12.24)	0.0016**
BUN	0.072(0.033, 0.11)	1.07 (1.03, 1.12)	0.0003***

CRP, C-reactive protein; NLR, neutrophil/lymphocyte ratio; A/G: Albumin/globulin; BUN: blood urea nitrogen; INR: international normalized ratio; CI, confidence interval. *p < 0.05, **p < 0.01, ***p < 0.001.

**Figure 3:**
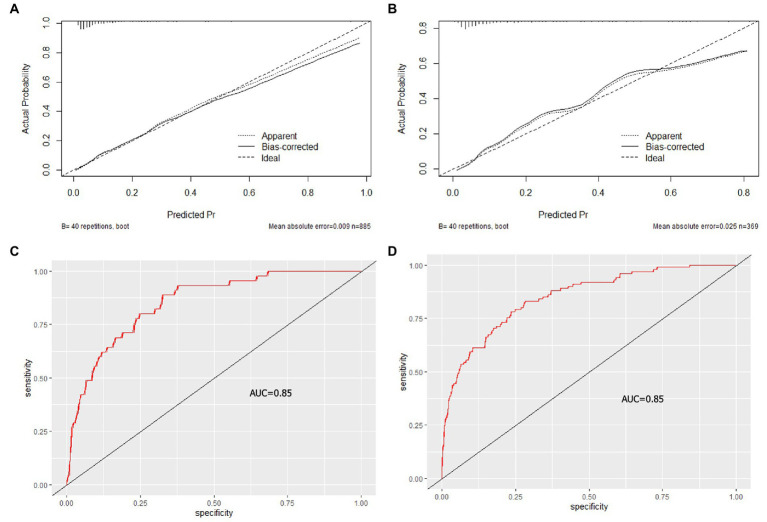
Calibration curve (top) and Receiver operating characteristic curves (bottom) of the nomogram. The calibration curve shows the locally estimated (solid line) smoothed observed probability versus estimated probability of deterioration events. The diagonal line (dashed line) shows ideal calibration. **(A)** training cohort, **(B)** testing cohort. The Receiver operating characteristic curves (red line) shows the performance to distinguish individuals with deteriorated COVID-19 from stable COVID-19 in the training cohort **(C)** and testing cohort **(D)**. AUC, area under the curve.

The predictive nomogram that integrated 5 selected features for the prediction of in-hospital clinical deterioration in the training cohort was shown in Figure 2C. The AUC of the nomogram was 0.85, which showed a good capability of discriminating individuals with clinical deterioration from stable COVID-19 patients (Figure 3C). Furthermore, the nomogram showed a superior overall net benefit within the wide and practical ranges of threshold probabilities evidenced by the DCA and CICA results (Figures 2D,E).

**Figure 4:**
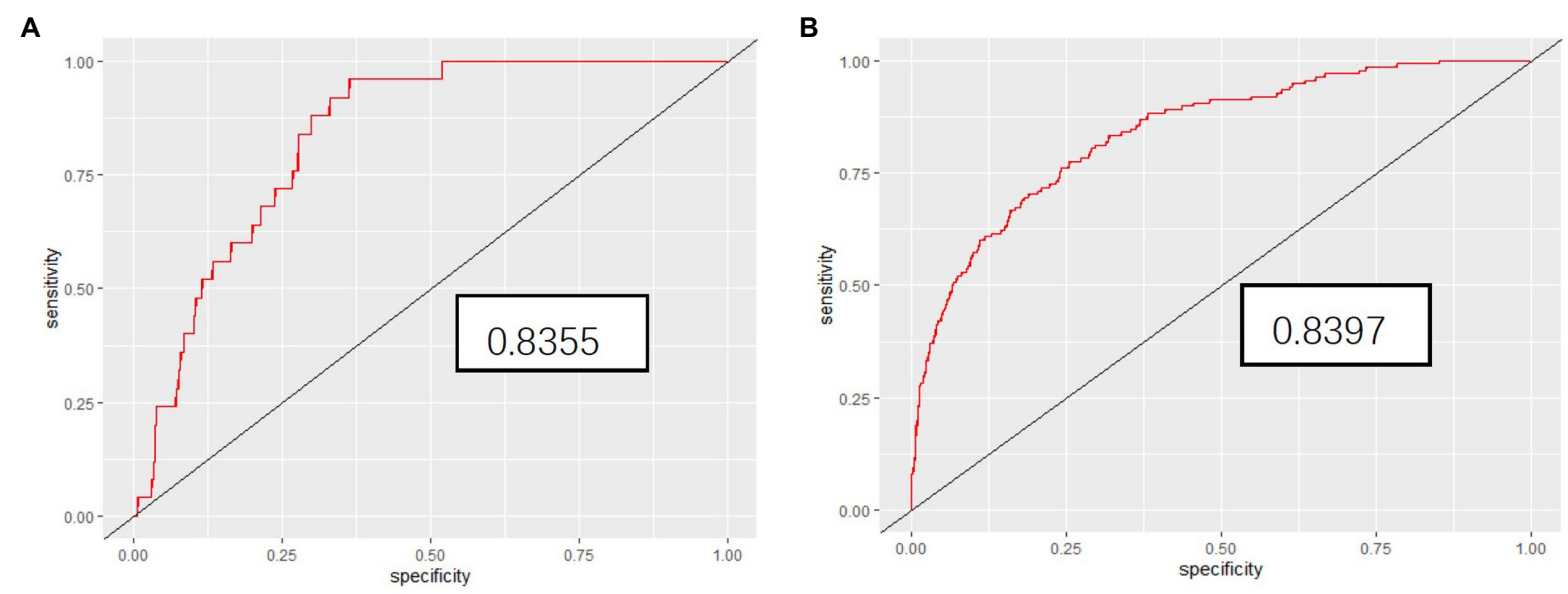
The Receiver operating characteristic curves (red line) shows the performance to distinguish individuals who needed ventilatory support **(A)** or those who died **(B)** using the five indicators.

The internal testing cohort included 370 patients (see Table 1). The AUC was 0.85 in predicting the deterioration rate (Figure 3D), indicating a good performance in differentiating the risk of progression in confirmed COVID-19 patients. When ventilation support or death was separately analyszed, the AUC of them was 0.8397 and 0.8355, respectively (Figures 4A,B).

### Calibration of the training cohort and testing cohort

The calibration plot for in-hospital clinical deterioration probability showed a good agreement between the prediction by nomograms and actual observation in the training cohort (Figure 3A) and validation cohort (Figure 3B), respectively.

## Discussion

In the present study, a prediction model on the outbreak of Omicron sub-variant BA.2 in Shanghai was built using clinical data from our hospital. It was found that high levels of CRP, NLR, INR, BUN, and decreased A/G at admission were significantly correlated with the probability of clinical deterioration. Using these 5 factors, an effective prognostic nomogram was constructed, which had a significantly high sensitivity and specificity to identify individuals with a high risk of deterioration. DCA and CICA further demonstrated the superiority of our nomogram evidenced by the net clinical benefit, which is invaluable for individualized assessment of in-hospital deterioration. To our best knowledge, this study is the first to build such a prediction model targeting the Omicron sub-variant BA.2. Other available models are based on clinical information of alpha or delta variant breaking out in 2020 or 2021.

From the baseline data, it can be seen that even the elderly in Shanghai showed mild to moderate symptoms although Omicron sub-variant BA.2 is so contagious. The deterioration rate is quite low, which is different from the outbreak of COVID-19 in Wuhan in 2020 (16). This might be attributed to the free vaccination among the public and strict quarantine policies in China (17, 18).

In the present study, continuous variables were used to build the prediction model, rather than categorical variables which are not always reliable and highly dependent on self-awareness. In contrast, laboratory tests are relatively accurate and readily available. High levels of CRP, NLR, INR, BUN, and low level of A/G are positively correlated with the deterioration rate. Compared with previous prediction models where age is always considered a major factor that influences the progression (4, 19, 20), our model did not take age into account. This is due to the observed characteristics in our study and a previous one (21), all patients were the elderly aged 65 or older. Initially, 1,830 patients were admitted to our hospital, but nearly all of the endpoint events occurred in elderly patients (age ≥ 65 years), only 3 patients had clinical deterioration among 575 patients younger than 65. In order to minimize the bias, we decided to focus on the elderly patients. Besides, age ≥ 65 is considered to be at highest risk for severe COVID-19 associated illness (22). In this elderly population, age is not an important factor that contributes to the endpoint events.

According to the previous research, the mechanism of COVID-19 infection is associated with inflammatory cytokine storm, oxidative stress, disseminated intravascular coagulation and distribution of ACE2 along the vascular endothelium, which lead to the multisystem dysfunction (23). Correlations between high levels of CRP and NLR and disease progression have been proven in many trials (24, 25), which have shown that Omicron BA.2 induces an inflammatory process evidenced by the activation of neutrophils and lymphocytes as well as the release of IL-6, CRP, and other inflammatory factors. INR is an index that reflects the functional status of the coagulation system. Increased INR values were significantly associated with COVID-19 severity and mortality (26). BUN reflects the function of the kidney, which has been validated as a significant risk factor of the disease severity (27). The reduced albumin-globulin ratio has been found to be a risk factor of COVID-19 severity in patients with cancer (28), and of great diagnostic significance in predicting the progression to severe disease states (29), which is consistent with our results.

## Strengths and limitations

Our study has a number of strengths: first, a large sample size and sufficient patients’ information guarantees the credibility of our conclusion. All of the included variables had no missing values and all the endpoints were collected during the study period. After the last confirmed COVID-19 patient was discharged, the hospital was closed for full disinfection. Second, our model is a practical quantitative prediction tool based on 5 features which are commonly used and easily obtained from routine blood tests. The performance of our nomogram is efficient for clinical practice. Third, the first-hand information of the Omicron outbreak in Shanghai in 2022 was of great practical use for medical practitioners in other countries where Omicron sub-variant BA.2 prevails.

The study also has a number of limitations. As a retrospective cohort study, we did not collect all the predictors that had been reported to be related to the progression of the disease, such as BMI, pro-BNP. Different clinical approaches were adopted at the early and late stages of this outbreak, full conformity to the recommended treatments is not guaranteed at different stages. Second, this is a single-centre study, and validation was performed in the same setting as the training cohort, an external validation would be much better to prove the effectiveness of the model. Furthermore, the construction of the model was based on hospitalized patients, our conclusion can not be extrapolated to the general population, but needs to be adjusted based on the different population characteristics.

## Conclusion

In summary, our data suggest that our nomogram can predict the risk of deterioration (requirement for ventilatory support or death) in elderly patients and it is clinically practical, which will facilitate medical decision making and allocating medical resources to those with critical conditions in order to reduce mortality.

## Data availability statement

The original contributions presented in the study are included in the article/supplementary material, further inquiries can be directed to the corresponding authors.

## Ethics statement

The studies involving human participants were reviewed and approved by the associated Ethics Committee of Shanghai Fourth People’s Hospital approved the study and waived the need for informed consent. The patients/participants provided their written informed consent to participate in this study.

## Author contributions

YB, YW, and ZY designed the initial concept. YB, YW, BX, JX, YH, HL, and ZY participated in acquisition and interpretation of data. YW and ZY performed the statistical analysis. YW, HL, and YB wrote the manuscript. All authors reviewed the manuscript.

## Conflict of interest

The authors declare that the research was conducted in the absence of any commercial or financial relationships that could be construed as a potential conflict of interest.

## Publisher’s note

All claims expressed in this article are solely those of the authors and do not necessarily represent those of their affiliated organizations, or those of the publisher, the editors and the reviewers. Any product that may be evaluated in this article, or claim that may be made by its manufacturer, is not guaranteed or endorsed by the publisher.
